# Long non-coding RNA MIDEAS-AS1 inhibits growth and metastasis of triple-negative breast cancer via transcriptionally activating NCALD

**DOI:** 10.1186/s13058-023-01709-1

**Published:** 2023-09-28

**Authors:** Dan Luo, Yiran Liang, Yajie Wang, Fangzhou Ye, Yuhan Jin, Yaming Li, Dianwen Han, Zekun Wang, Bing Chen, Wenjing Zhao, Lijuan Wang, Xi Chen, Liyu Jiang, Qifeng Yang

**Affiliations:** 1 Department of Breast Surgery, General Surgery, Qilu Hospital, Cheeloo College of Medicine, Shandong University, Wenhua Xi Road No. 107, Jinan, 250012 Shandong China; 2 Pathology Tissue Bank, Qilu Hospital of Shandong University, Jinan, 250012 Shandong China; 3 Research Institute of Breast Cancer, Shandong University, Jinan, 250012 Shandong China

**Keywords:** Triple-negative breast cancer, MIDEAS-AS1, MATR3, NCALD, Prognosis

## Abstract

**Background:**

Triple-negative breast cancer (TNBC) is a subtype of breast cancer with higher aggressiveness and poorer outcomes. Recently, long non-coding RNAs (lncRNAs) have become the crucial gene regulators in the progression of human cancers. However, the function and underlying mechanisms of lncRNAs in TNBC remains unclear.

**Methods:**

Based on public databases and bioinformatics analyses, the low expression of lncRNA MIDEAS-AS1 in breast cancer tissues was detected and further validated in a cohort of TNBC tissues. The effects of MIDEAS-AS1 on proliferation, migration, invasion were determined by in vitro and in vivo experiments. RNA pull-down assay and RNA immunoprecipitation (RIP) assay were carried out to reveal the interaction between MIDEAS-AS1 and MATR3. Luciferase reporter assay, Chromatin immunoprecipitation (ChIP) and qRT-PCR were used to evaluate the regulatory effect of MIDEAS-AS1/MATR3 complex on NCALD.

**Results:**

LncRNA MIDEAS-AS1 was significantly downregulated in TNBC, which was correlated with poor overall survival (OS) and progression-free survival (PFS) in TNBC patients. MIDEAS-AS1 overexpression remarkably inhibited tumor growth and metastasis in vitro and in vivo. Mechanistically, MIDEAS-AS1 mainly located in the nucleus and interacted with the nuclear protein MATR3. Meanwhile, NCALD was selected as the downstream target, which was transcriptionally regulated by MIDEAS-AS1/MATR3 complex and further inactivated NF-κB signaling pathway. Furthermore, rescue experiment showed that the suppression of cell malignant phenotype caused by MIDEAS-AS1 overexpression could be reversed by inhibition of NCALD.

**Conclusions:**

Collectively, our results demonstrate that MIDEAS-AS1 serves as a tumor-suppressor in TNBC through modulating MATR3/NCALD axis, and MIDEAS-AS1 may function as a prognostic biomarker for TNBC.

**Supplementary Information:**

The online version contains supplementary material available at 10.1186/s13058-023-01709-1.

## Background

Breast cancer is one of the most common malignant tumors that has serious effects on the health of women worldwide [1, 2]. Triple-negative breast cancer (TNBC), one subtype of breast cancer, lacks estrogen receptor (ER), progesterone receptor (PR), and expresses low levels of human epidermal growth factor receptor 2 (HER-2) and accounts for approximately 15–20% of all breast carcinomas [3–5]. TNBC is characterized by higher rates of relapse, greater metastatic potential, and shorter overall survival compared with other major breast cancer subtypes [4, 5]. Therapeutic methods for TNBC patients usually include surgery, chemotherapy, radiotherapy, and immunotherapy [6–9]. Clinically, although significant progress in the treatment of TNBC over the last decade, recurrence and metastasis remain the principal causes of mortality in patients with this disease [10, 11]. Therefore, elucidating the potential molecular mechanisms underlying TNBC progression is of great significance to provide promising novel treatment targets and prognostic biomarkers.

In recent years, the potential role of long non-coding RNAs (lncRNAs) has attracted increasing attention in different kinds of cancers. LncRNAs are commonly defined as a class of RNA molecules with a length of more than 200 nucleotides and little or no coding capacity [12]. LncRNAs are abnormally expressed in many tumors and closely associated with prognosis of tumor patients [13]. Various studies reported that lncRNAs are responsible for human tumorigenesis and cancer progression by functioning either as oncogenes or tumor suppressors [14–16] and play pivotal roles in tumor cell growth, differentiation, invasiveness, metastasis, anti-apoptosis, and drug resistance [17–19]. For example, long non-coding RNA SNHG12 promotes tumor progression and sunitinib resistance in renal cell carcinoma [20]. LncRNA CASC2 inhibits hypoxia-induced pulmonary artery smooth muscle cell proliferation and migration by regulating the miR-222/ING5 axis [21]. Previous reports have shown that lncRNAs are involved in the regulation of gene expression through a multitude of mechanisms depending on their subcellular localization. The cytoplasmic lncRNAs are most commonly reported to function as competing endogenous RNAs (ceRNA) to regulate gene expression. For example, long non-coding RNA NEAT1 promotes ferroptosis by modulating the miR-362-3p/MIOX axis as a ceRNA [22]. LINC00673 is activated by YY1 and promotes the proliferation of breast cancer cells via the miR-515-5p/MARK4/Hippo signaling pathway [23]. On the other hand, the nuclear lncRNAs could regulate gene expression by participating in several biological processes, such as chromatin organization, nuclear structure organization, severing as transcriptional and post-transcriptional regulators, and acting as scaffolds for TFs [24, 25]. Given the abundant quantities and diversified regulatory mechanisms of lncRNAs, more efforts are needed to better understand the biological functions and molecular mechanisms of lncRNAs in TNBC and provide information for improving the treatment and prognosis of TNBC.

In the present study, we aimed to explore the function and underlying mechanisms of a novel identified lncRNA, MIDEAS-AS1, in TNBC. We discovered that MIDEAS-AS1 were markedly reduced in breast cancer according to GEO and TCGA databases, which was further confirmed in our cohort. Further, we compared the expression of MIDEAS-AS1 in distinct subtypes in breast cancer, and MIDEAS-AS1 was significantly decreased in TNBC. Moreover, low MIDEAS-AS1 expression was associated with poor prognosis of patients with TNBC. Using in vitro and in vivo experiments, we further demonstrated that MIDEAS-AS1 could inhibit the progression and metastasis of TNBC through interacting with MATR3 to upregulate the transcription of NCALD and subsequently inhibited the NF-κB signaling pathway. These findings indicated that MIDEAS-AS1 might function as a tumor-suppressor lncRNA with potential as a diagnostic/prognostic marker and may offer a novel target for the treatment of patients with TNBC.

## Materials and methods

### Tissue samples

Human breast cancer tissues and adjacent non-tumor tissues were obtained from patients at the Qilu Hospital. Written informed consent was provided by all patients, and the study was achieved approval by the Ethical Committee on Scientific Research of Shandong University Qilu Hospital (IRB number, KYLL-2016(KS)-140).

### Cell cultures

All cell lines were bought from American Type Culture Collection (ATCC, Manassas, VA). MDA-MB-231, MDA-MB-468 and HEK293T cells were cultured with DMEM. The above media contained 100 U/ml penicillin, 100 μg/ml streptomycin, 10% fetal bovine serum (Cell-Box, HK, China) and were cultured with 5% CO_2_ in a humidified cell-culture incubator at 37 °C.

### Breast cancer organoids

Tissues obtained was minced and then, placed into 50 ml conical tube containing 20 ml Advanced DMEM/F-12 (Gibco, CA, USA) which was supplemented with 1× Glutamax, 10 mM HEPES (Invitrogen, Texas, USA), 1 mg/ml BSA (BasalMedia, Shanghai, China), 1× Primocin (InvivoGen, Texas, USA) and 1 mg/ml collagenase (Sigma-Aldrich, MO, USA), 0.01 mg/ml Hyaluronidase (Sigma-Aldrich, MO, USA), 10 μmol Y-27632 (Sigma-Aldrich, MO, USA). Tubes containing minced tissue and collagenase were digested on a 37 °C constant temperature shaker for about 1.5 h. After digestion, filter with 100 μm cell strainer filtration, cell filtrate was enriched by centrifugation at 300 g, 4 °C, at low-speed horizontal-refrigerated centrifuge and washed with cold Advanced DMEM/F-12 twice. Isolated cells were uniformly mixed with Cultrex RGF Basement Membrane Extract Type 2 (R&D Systems, MN, USA). A 50 μl drop of this suspension was placed in center of a well in an uncoated 48 well plate and allowed to harden for 30 min at 37 °C. Finally, each well was added 350 μl of human organoid medium contained Advanced DMEM/F-12 which was supplemented with 1× Primocin (Invitrogen, Texas, USA), 10 mM HEPES, 1× Glutamax, 1× B27 (Gibco, CA, USA), 100 ng/ml Nrg1 (PeproTech, NJ, USA), 100 ng/ml Noggin (PeproTech, NJ, USA), 50 ng/ml EGF (PeproTech, NJ, USA),500 ng/ml Human R-spondin-1 (PeproTech, NJ, USA), 500 nM A83-01 (Tocris Bioscience, MN, USA),5 μmol Y-27632, 5 mM Nicotinamide (Sigma-Aldrich, MO, USA), 3 μmol SB202190 (Sigma-Aldrich, MO, USA), 10 nM Prostaglandin E2 (Sigma), 1.25 mM n-Acetyl Cysteine (Sigma-Aldrich, MO, USA). Organoids were maintained in 37 °C humidified atmospheres under 5% CO_2_. Medium was changed every 3–4 days, and organoids were passaged using TrpLE Express (Invitrogen, Texas, USA) approximately every 2–4 weeks.

### RNA sequencing analysis

Breast cancer gene expression data were downloaded from TCGA (https://tcga-data.nci.nih.gov/tcga) and GEO (http://www.ncbi.nlm.nih.gov/geo) database. The data analysis was performed with R software using the DEGseq package. The threshold set for differences was |log2(Fold change) |> 1 and *p*-value < 0.05.

### Cell transfection

The small interfering RNAs targeting MIDEAS-AS1, MATR3, NCALD and the scrambled oligonucleotides (NC; 20–50 nM) were purchased from GenePharma (Shanghai, China). Full sequence of MIDEAS-AS1, MATR3, NCALD complementary DNA (cDNA) was amplified by PCR and then, inserted into the vector pcDNA3.1-GFP (abbreviated as “pcDNA3.1”) or pEnter (WZ Biosciences, Jinan, China) to construct pcDNA3.1/MIDEAS-AS1, pcDNA3.1/NCALD, pEnter/MATR3 plasmids. Empty vector (pcDNA3.1, pEnter) was regarded as the control. Using Lipofectamine 2000 (Invitrogen, Texas, USA), all vectors were transfected into breast cancer cells. After 24 h of transfection, cells were collected for following experiments. The sequences that were used are shown in Additional file 1: Table S1.

### Quantitative real-time PCR (qRT-PCR)

Total RNA was extracted using the RNA-easy Isolation Reagent Kit (Vazyme, Nanjing, China). Reverse transcription from 1 μg RNA to cDNA was performed using the PrimeScript reverse transcriptase reagent kit (Takara, Shiga, Japan). Real-time PCR was performed with SYBR qPCR SuperMix Plus (novoprotein, Suzhou, China) on a QuantStudio 6 Flex Real-Time PCR System. Results were analyzed using the comparative Ct method normalizing to Actin. The primer sequences are shown in Additional file 1: Table S2.

### Subcellular fractionation

Separation of nuclear and cytosolic fractions was performed using the PARIS Kit (Invitrogen, Texas, USA) according to the manufacturer’s instructions. Afterward, MIDEAS-AS1, GAPDH (cytoplasmic control) and U6 (nuclear control) in a cytoplasmic fraction or nuclear fraction were detected by qRT-PCR.

### RNA immunoprecipitation (RIP)

The RIP experiments were performed strictly with a Magna RIP RNA-Binding Protein Immunoprecipitation kit (Millipore, Burlington MA, USA) according to the manufacturer’s instruction. Each RIP reaction required 100 μl of 1 × 10^7^ MDA-MB-231 cell lysate, and each immunoprecipitation required 5 μg of antibody. The expression of MIDEAS-AS1 in the precipitated of anti-MATR3 and negative control (IgG) was detected by qPCR, and the content in the IgG precipitate was used as a reference. qRT-PCR was performed as described above.

### Cell migration and invasion assay

After transfection treatment for 24 h, MDA-MB-231 and MDA-MB-468 were harvested and resuspended in serum-free DMEM medium and seeded into the upper transwell chambers containing 8 μm pores. As for invasion experiment, the cells were seeded in Matrigel matrix-plated chambers. Culture medium supplemented with 20% FBS was added to the lower chamber. After incubation for 20 h for migration and 24 h for invasion at 37 °C, the chambers were fixed with methanol, stained with 0.5% crystal violet. Then, the cells on the upper surface were wiped off and allowed to dry at room temperature. The migrated and invasive cells were counted and photographed under a light microscopy (200×) (Olympus, Tokyo, Japan).

### Wound healing experiment

The cells were seeded in 24-well plates at a density of 3.5 × 10^5^ cells per well for MDA-MB-231 and 5 × 10^5^ cells per well for MDA-MB-468. Then, incubated the wells with cell culture medium at 37 °C overnight. When the cells at approximately 90% confluence, an artificial wound was made with a 10-µl sterile pipette tip. The fragments were washed thoroughly with PBS, and the wells were added the serum-free medium, cultured for various amounts of time. MDA-MB-231 were incubated for 24 h, and MDA-MB-468 cells were incubated for 48 h. We used a microscope to detect cell migration near the wound and obtain images. The images were processed using ImageJ software to quantify the open wound area as average open wound area % and a histogram was drawn.

### Fluorescence in situ hybridization (FISH)

FISH was performed using the RNA FISH Probe Mix Kit (GenePharma, Shanghai, China) according to the manufacturer’s protocol. Briefly, we placed the cell slides in a 24-well plate, seeded the cells at a density of 1.5 × 10^5^ cells per well, and incubated overnight at 37 °C. The medium was discarded and washed twice with PBS in each well, and 4% paraformaldehyde was added and fixed at the room temperature for 15 min. After blocking, the cells were incubated with the lncRNA probes at 37 °C for 16 h and washed three times with a washing solution for 15 min. Subsequently, we added Hoechst 33342 (Beyotime, Suzhou, China) fluorescent dye solution for 10 min at the room temperature while avoiding light and then, observed the distribution of MIDEAS-AS1 under the Confocal Microscope ZEISS LSM 880 (ZEISS, Berlin, Germany).

### Chromatin immunoprecipitation (ChIP)-qPCR assay

ChIP assays were performed using a ChIP assay kit (Cell Signaling Technology, MA, USA) according to the manufacturer’s instructions. Briefly, cells were fixed for 10 min with 1% free formaldehyde and then disrupted in SDS lysis buffer. Chromatin was sonicated by Bioruptor® Pico (Diagenode, Belgium) to shear DNA to an average length ranging from 200 to 1000 bp, as verified by agarose gel electrophoresis. Next, chromatin was immunoprecipitated with anti-Flag (Cell signaling Technology, USA), and normal rabbit IgG was used as the negative control. Final DNA extractions were quantitative-PCR amplified using primer pairs that cover the sequence in the NCALD promoter region (−2000 bp to + 100 bp).

### RNA–protein pull-down assays

In vitro transcription of sense, antisense or truncated MIDEAS-AS1 was achieved by T7 RNA polymerase (Thermo Fisher, MA, USA). Subsequently, the product of in vitro transcription was obtained biotin-labeled with PierceTM RNA 3’ End Biotinylation Kit (Thermo Fisher, MA, USA). Washed streptavidin magnetic beads were incubated with 50 pmol of purified biotinylated transcripts at room temperature for 30 min, followed by addition of the whole-cell lysates (20–200 μg) from MDA-MB-231 cells and incubated for 1 h at 4 °C. The beads containing DNA and proteins were then washed and eluted, then beads were boiled, and precipitated protein was separated by SDS-PAGE and detected by Western blotting analysis.

### Western blotting

Protein samples were harvested from cell lysates, and the concentration of total protein was measured with a BCA Protein assay kit (Millipore, Burlington MA, USA). Protein were separated by SDS-PAGE and transferred to a PVDF membrane (Millipore, Burlington MA, USA). The membrane was blocked with 5% nonfat milk at room temperature and incubated overnight with specific primary antibodies at 4 °C: rabbit anti-Fibronectin (Proteintech, Wuhan, China), rabbit anti-N-cadherin (Proteintech, Wuhan, China), rabbit E-cadherin (Proteintech, Wuhan, China), rabbit anti-Vimentin (Proteintech, Wuhan, China), rabbit anti-MMP-9 (Cell Signaling Technology, MA, USA), mouse anti-GAPDH (Servicebio, Wuhan, China), rabbit anti-MATR3 (Proteintech, Wuhan, China), mouse anti-Actin (Servicebio, Wuhan, China), rabbit anti-NCALD (Proteintech, Wuhan, China), rabbit anti-TGF-β (Proteintech, Wuhan, China), rabbit anti-p-p65 (Affinity Biosciences, OH, USA), mouse anti-p65 (Proteintech, Wuhan, China), rabbit anti-p-Erk1/2 (Cell Signaling Technology, MA, USA), rabbit anti-Erk1/2 (Proteintech, Wuhan, China), washed with Tween-20/TBS and incubated with horseradish peroxidase-conjugated secondary antibodies for 2 h, followed by enhanced chemiluminescence detection (Vazyme, Nanjing, China), and protein bands were visualized using a ECL detection system (Tanon, Shanghai, China), band density was determined using the ImageJ analyzer software (version 1.48).

### Immunofluorescence analysis

The cell coverslips were placed in a 24-well plate, seeded transfected MDA-MB-231 at a density of 1.5 × 10^5^ cells per well, and incubated overnight at 37 °C. Then, the cells were fixed with 4% paraformaldehyde for 15 min at room temperature and blocked with 10% goat serum for 30 min. The cells were incubated with rabbit anti-NCALD (Proteintech, Wuhan, China) at 4 °C overnight. Cells were washed three times in PBS and incubated for 1 h at room temperature with FITC conjugated-goat anti-rabbit antibody (ZSGB-BIO, Beijing, China). After several washes, the cells were added hoechst 3342 (Beyotime, Suzhou, China) fluorescent dye solution, and coverslips were mounted to the slides using fluorescent mounting medium (PROLONG-GOLD, Thermo Fisher Scientific, MA, USA). Coverslips were imaged on the Nikon Eclipse Ti microscope (Nikon, Tokyo, Japan).

### Cell proliferation assay

1.5 × 10^3^ transfected cells were seeded into each well of five 96-well plates. The cells were cultured for five consecutive days and added with 20 μl of 3-(4,5-dimethylthiazol-2-yl)-2,5-diphenyltetrazolium bromide (MTT, Beyotime, Suzhou, China). Afterward, the cells were maintained at 37 °C, 5% CO_2_ for another 4 h, and the MTT solution was removed, and 100 μl DMSO was added to each well. The 490-nm optical absorption value of each well was obtained, and the proliferation curves were established accordingly by using the GraphPad Prism 8.3.0 software.

### Colony formation assay

In total, 500 cells were plated in a six-well plate and cultured in DMEM medium containing 10% FBS. Medium was changed every three days. MDA-MB-231 cells were cultured for two weeks, and MDA-MB-468 cells were cultured for four weeks. Then, cells were washed once with PBS solution and fixed with methanol for 15 min, and 500 μl of 0.5% crystal violet (Beyotime, Suzhou, China) was added to each well for 30 min. The colonies were imaged and counted.

### Cell cycle analysis

Cell cycle analysis was performed using Cell Cycle Staining Buffer (Multi Sciences, Hangzhou, China) following the manufacturer’s protocol. Briefly, transfected cells were digested and washed, and then, resuspended in 500 μl of cell cycle staining buffer for 15 min. The cells were examined on a FACSCalibur (BD Biosciences, CA, USA) within 1 h.

### Cell apoptosis assay

For apoptosis analysis, FITC-Annexin-V/7-AAD double staining method was used (BD Biosciences, CA, USA). Transfected cells were collected, washed twice with cold PBS, centrifuged at 1000 rpm 5 min, and the supernatant discarded. Then, resuspended cells in 1× Binding buffer at a concentration of 1 × 10^6^ cell/ml. Transfer 100 μl of solution to a 1.5 ml culture tube, added 5 μl FITC-Annexin V, 5 μl 7-AAD to resuspend the cells, reacted for 15 min at the room temperature in the dark. After staining, added 400 μl of 1× Binding buffer to each tube, the apoptosis percentage were analyzed by FACSCalibur flow cytometer (BD) within 1 h.

### Luciferase reporter assay

Sequences containing all length or truncated of NCALD promoter were subcloned into pGL4.26-control vector (Addgene, MA, USA). For the reporter assay, HEK-293 T cells were plated onto 48-well plates, and the pGL4.26-NCALD or pGL4.26-truncated-NCALD, the Renilla luciferase plasmid (pRL-TK) were contransfected with pcDNA3.1, MIDEAS-AS1 overexpression plasmids, si-NC, si-MATR3 using Lipofectamine 2000. Finally, each group of cells was transfected with. After transfection for 48 h, the cells were harvested and assayed with a luciferase reporter assay system (Promega, Madison, WI, USA) according to the manufacturer’s instructions. The results were normalized against Renilla luciferase activity.

### Xenograft tumor formation assay

BALB/c nude mice (female, 4-week-old) with weights of about 20 g were procured from GemPharmatech (Jiangsu, China) for in vivo study. The transfected cells were injected subcutaneously into mice, and the width and length of formed tumors were monitored every 5 days after injection. Tumor volumes were calculated using the following formula: tumor volume = length × width^2^ × 0.5. Following 25 days, the nude mice were sacrificed, and the collected tumors were weighed and then, fixed in formalin for immunohistochemical (IHC) staining and Hematoxylin and eosin (H&E) staining. To evaluate the influence of MIDEAS-AS1 on metastasis, the transfected cells were injected into the lateral tail veins of nude female mice (six mice per group). After about 2 months, the mice were sacrificed, and the lungs were collected to evaluate the number of pulmonary metastatic lesions and then, also were fixed for HE staining. The animal experiments were approved by the Ethical Committee on Scientific Research of Shandong University Qilu Hospital.

### Immunohistochemistry

Paraffin-embedded tissues sections were deparaffinized and rehydrated with xylene, gradient ethanol (70, 80, 95, 100%) and incubated for 20 min in 3% H_2_O_2_ to block endogenous peroxidase activity. After blocking with 10% goat serum and then incubated with 50 μl rabbit anti-Ki67 (Proteintech, Wuhan, China), rabbit anti-MATR3 (Proteintech, Wuhan, China), rabbit anti-NCALD (Proteintech, Wuhan, China) at 4 °C overnight. Subsequently, sections were incubated with an appropriate secondary antibody for 20 min at 37 °C and developed with diaminobenzidine and stained with hematoxylin for 5 min. After each treatment, the sections were dehydrated, cleared, mounted, and viewed under the microscope.

### Statistical analysis

Statistical test in this study was performed using GraphPad Prism software 8.3.0 software. Results were expressed as the mean ± standard deviation (SD). The Kaplan–Meier method and log-rank test were used to analyze survival rate. Differences of two or more groups were analyzed using the student’s t-test or one-way/two-way ANOVA. Statistical significance was determined as p < 0.05. All tests were conducted in triplicates.

## Results

### MIDEAS-AS1 is downregulated in TNBC tissues and low MIDEAS-AS1 level predicts poor prognosis of TNBC

**Fig. 1:**
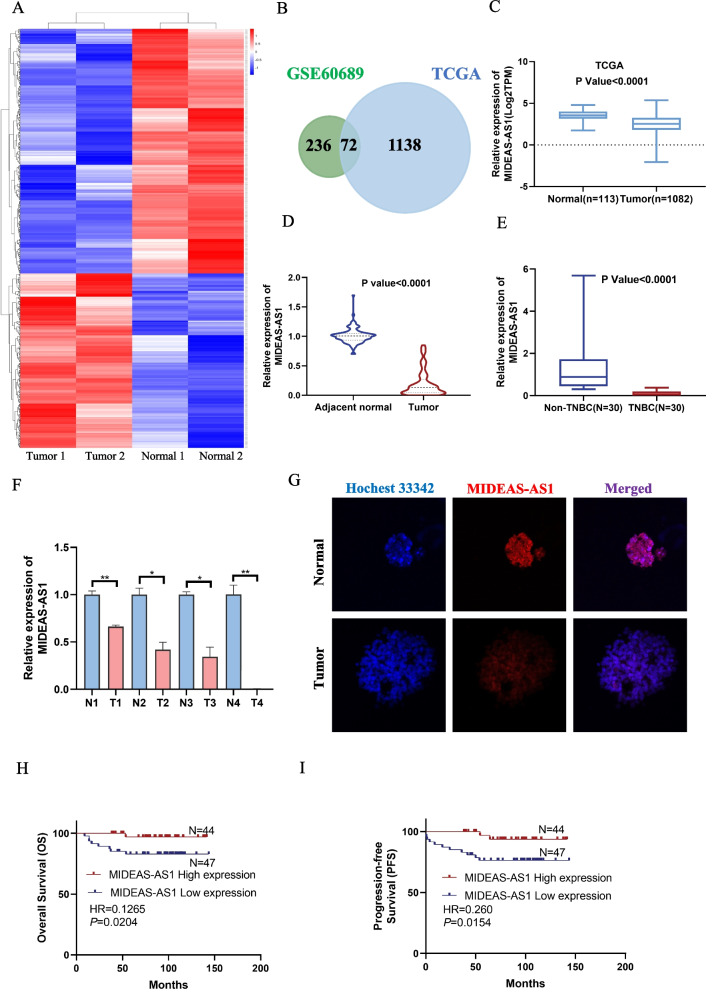
LncRNA MIDEAS-AS1 is downregulated in TNBC tissues and is associated with poor progression. **A** Heat map shows the significantly expressed lncRNAs in breast cancer samples compared to normal tissues from GEO (GSE60689) databases. The red shades represent high expression, and blue shades represent low expression. **B** Overlapping lowly expressed lncRNAs identified in GEO and TCGA lncRNA databases. **C** TCGA database showed that MIDEAS-AS1 was abnormally low expressed in breast cancer tissues. **D** The mRNA expression of MIDEAS-AS1 was lower in breast cancer tissues than in adjacent tissue. **E** qRT-PCR analysis of the expression of MIDEAS-AS1 in TNBC tissues and non-TNBC tissues. **F** qRT-PCR analysis of the relative expression of MIDEAS-AS1 in breast cancer organoids compared to the normal. **G** FISH analysis of the MIDEAS-AS1 in breast cancer and normal organoids. Scale bars, 20 μm. **H** Kaplan–Meier analysis showed the association between MIDEAS-AS1 expression and overall survival of TNBC patients. **I** Kaplan–Meier analysis showed the association between MIDEAS-AS1 expression and progression-free survival (PFS) of TNBC patients. Data were shown as mean ± SD. (*p < 0.05, ** *p* < 0.01, *** *p* < 0.001)

To explore the potential role of lncRNAs in TNBC, we first analyzed the differentially expressed lncRNAs using GEO (Fig. 1A) and TCGA databases (Additional file 1: Fig. S1A). Then, integrated analysis was performed on the lowly expressed lncRNAs at the GSE60689 and TCGA lncRNA databases (Fig. 1B). Among them, the newly named lncRNA MIDEAS-AS1 attracted our attention. A search of the UCSC database (http://genome.ucsc.edu/) and CPC2 database (http://cpc2.gao-lab.org) revealed that MIDEAS-AS1 was located on the human chromosome 14q24.3 and had three exons, without protein-coding capabilities (Additional file 1: Fig. S1B-C). Based on TCGA datasets, we validated that MIDEAS-AS1 was markedly low expressed in breast cancer tissues compared to adjacent normal tissues (Fig. 1C). Additionally, we also analyzed the expression levels of MIDEAS-AS1 in different subtypes of breast cancer. Among patients with breast cancer, the expression of MIDEAS-AS1 was lower in TNBC than in other subtypes through the GSE21653 database (Additional file 1: Fig. S1D). To further validate the decrease in MIDEAS-AS1 in breast cancer, we examined the expression of MIDEAS-AS1 using qRT-PCR in breast cancer tissues and their adjacent normal breast tissues, as well as, TNBC and non-TNBC tissues. Compared to the adjacent normal tissues, the expression of MIDEAS-AS1 was significantly downregulated in breast cancer tissues (Fig. 1D). Meanwhile, we also confirmed the results that patients with TNBC had a significantly lower expression of MIDEA-AS1, compared with non-TNBC (Fig. 1E). Moreover, we further examined the MIDEAS-AS1 expression level in breast cancer organoids and normal organoids, and the result revealed that MIDEAS-AS1 was significantly low expressed in the breast cancer organoids (Fig. 1F). In addition, fluorescence in situ hybridization (FISH) assay revealed similar results, and downregulated expression of MIDEAS-AS1 was identified in breast cancer organoids compared to the normal organoid (Fig. 1G). There were some literatures reported that MIDEAS-AS1 was associated with tumor stage and tumor-node-metastasis (TNM) stage [26]. To assess the clinical significance of MIDEAS-AS1 in TNBC, we analyzed the relationship between MIDEAS-AS1 expression level and clinicopathological characteristics. We found that the low expression level of MIDEAS-AS1 was significantly correlated with larger tumor size and higher pathological grade (Table1). Moreover, Kaplan–Meier survival analysis showed that lower expression level of MIDEAS-AS1 was correlated with significantly poorer overall survival (OS) rate (Fig. 1H) and progression-free survival (PFS) rate of patients with TNBC (Fig. 1H-I). Together, these data indicated that MIDEAS-AS1 was downregulated in TNBC and low expression of MIDEAS-AS1 was associated with poor prognosis of TNBC patients.

**Table 1:** Correlation between MEDIA-AS1 mRNA expression and clinicopathological characteristics in 91 triple-negative breast cancer patients

Characteristics	Total cases (*n* = 91)	MEDIA-AS1 expression level		
		Low	High	*p* value
Age				
≤ 50	41	24	17	
> 50	50	23	27	0.233772
Menopausal state				
Pre	30	16	14	
Post	57	29	28	0.827498
N/A	4	2	2	
Pathological grade				
≤ 2	51	21	30	
> 2	39	26	13	**0.016440**^a^
N/A	1	0	1	
Ki67				
Low	12	4	8	
High	79	43	36	0.173006
Tumor size				
≤ 2	37	14	23	
> 2	54	33	21	**0.029093**^a^
Lymphatic metastasis				
Positive	34	18	16	
Negative	57	29	28	0.848837
N/A	1	0	1	
Metastasis status				
Positive	13	7	6	
Negative	63	31	32	0.760652
N/A	15	9	6	

The bold values represent significant difference (*P* < 0.05)

^a^* *p* < 0.05; ** *p* < 0.01; *** *p* < 0.001

### MIDEAS-AS1 reduced the proliferation, migration and invasion of TNBC cells in vitro

**Fig. 2:**
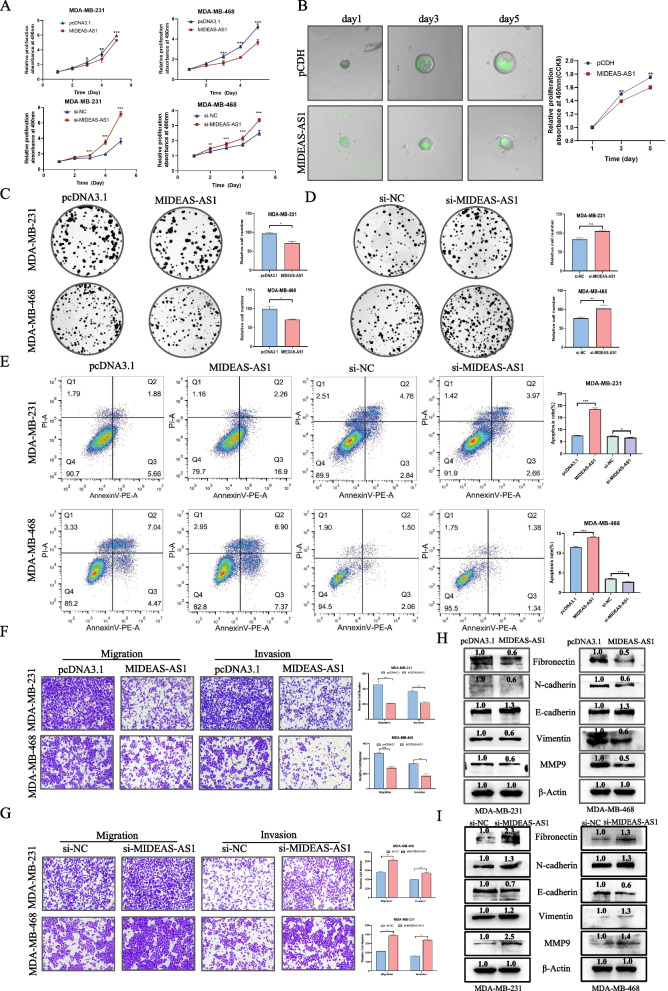
MIDEAS-AS1 inhibits TNBC cell proliferation and migration in vitro. **A–D** The effects of MIDEAS-AS1 overexpression and knockdown on the proliferation were examined by MTT assay (**A-B**) and colony formation assays (**C-D**). **E** Flow cytometry was performed to measure the effect of MIDEAS-AS1 on apoptosis. **F-G** Transwell and invasion assays were used to evaluate the motility of MDA-MB-231 and MDA-MB-468 cells transfected with MIDEAS-AS1-overexpressing vector or control vector (**F**) and si-NC or si-MIDEAS-AS1 (**G**). **H** Western blot analysis of EMT-related proteins in MDA-MB-231 and MDA-MB-468 cells after overexpression of MIDEAS-AS1. **I** Western blot analysis of EMT-related proteins in MDA-MB-231 and MDA-MB-468 cells after knockdown of MIDEAS-AS1. Data were shown as mean ± SD. (*p < 0.05, ** *p* < 0.01, *** *p* < 0.001)

To explore the potential roles of MIDEAS-AS1, we transfected the MIDEAS-AS1 overexpression plasmids into MDA-MB-231 and MDA-MB-468 cells, and the overexpression efficiency was determined by qRT-PCR (Additional file 1: Fig. S2A, S2B). Results from MTT assay indicated that the proliferation ability of MDA-MB-231 and MDA-MB-468 cells with MIDEAS-AS1 overexpression was reduced compared to control group (Fig. 2A). Meanwhile, overexpression of MIDEAS-AS1 also inhibited the proliferation of breast cancer organoids (Fig. 2B). Moreover, overexpression of MIDEAS-AS1 repressed cell colony-forming activity (Fig. 2C-D). Additionally, flow cytometry was used to detect the apoptosis rate and cell cycle after different treatments. Our results indicated that MIDEAS-AS1 overexpression led to remarkably increased apoptosis rate (Fig. 2E) and cell number in G1 phase (Additional file 1: Fig. S2C). On the other hand, MIDEAS-AS1 knockdown promoted the proliferation and colony-formation abilities of TNBC cells (Fig. 2A, D). Furthermore, the apoptosis rate was obviously decreased and the number of cells in G1 phase was less in MIDEAS-AS1 knockdown group compared to that in control group (Fig. 2E, Additional file 1: Fig. S2C). We then investigated the effect of MIDEAS-AS1 on TNBC cell migration and invasion using transwell assays and wound healing assay in MDA-MB-231 and MDA-MB-468 cells. The results of transwell assays showed a significant reduction in the migratory and invasive abilities of MDA-MB-231 and MDA-MB-468 cells after MIDEAS-AS1 overexpression (Fig. 2F). Furthermore, wound healing assay indicated that the wound closure area of MDA-MB-231 and MDA-MB-468 cells was dramatic decreased following MIDEAS-AS1 overexpression (Additional file 1: Fig. S2D). Consistently, MIDEAS-AS1 knockdown led to increased migratory and invasive abilities of TNBC cells (Fig. 2G). Given that epithelial-mesenchymal transition (EMT) is one of the major mechanisms for cancer metastasis, we further examined the effect of MIDEAS-AS1 on the expression of EMT-related marker proteins by Western blot. The results showed that N-cadherin, vimentin, fibronectin and MMP9 proteins were downregulated, while the E-cadherin protein was upregulated in MDA-MB-231 and MDA-MB-468 cells after overexpression of MIDEAS-AS1 (Fig. 2H). On the contrary, N-cadherin, vimentin, fibronectin and MMP9 proteins were upregulated, and the E-cadherin protein was downregulated in TNBC cells after MIDEAS-AS1 knockdown (Fig. 2I). Therefore, MIDEAS-AS1 could regulate the EMT process to modulate TNBC metastasis. Collectively, these results indicated that MIDEAS-AS1 played critical roles in suppressing cell proliferation and mobility of TNBC cells.

### MIDEAS-AS1 directly interacts with MATR3 to carry out its function

**Fig. 3:**
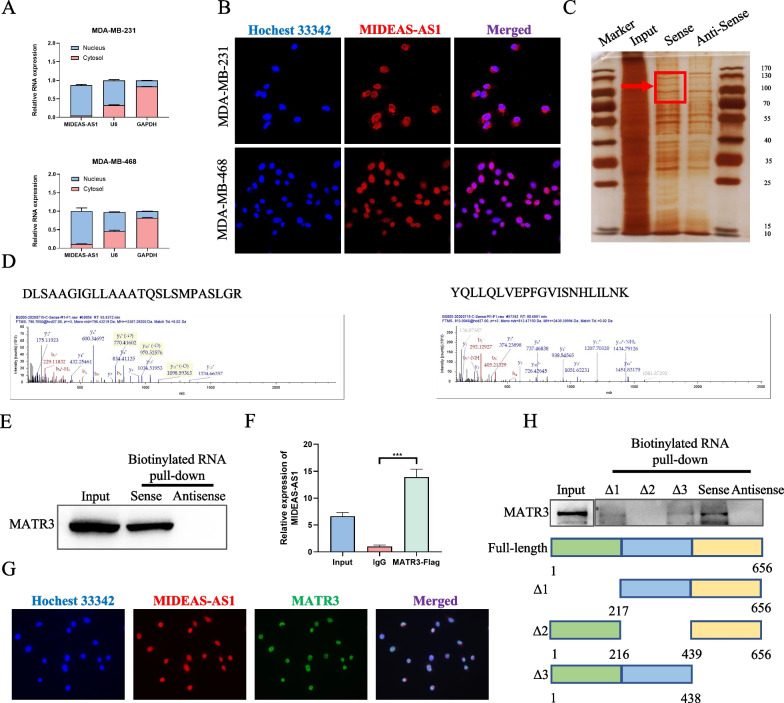
MIDEAS-AS1 interacts with MATR3. **A** The expression level of MIDEAS-AS1 in the subcellular fractions of MDA-MB-231 and MDA-MB-468 cells was detected by qRT-PCR, with U6 and GAPDH as nuclear and cytoplasmic markers, respectively. **B** FISH analysis of the location of MIDEAS-AS1 (red) in the cytoplasm and nuclear fractions of MDA-MB-231 and MDA-MB-468 cells. Scale bars, 20 μm. **C** Biotin-labeled MIDEAS-AS1 was incubated with MDA-MB-231 cell lysates for pull-down, followed by SDS-PAGE separation, silver staining. **D** Peptide sequences analysis interacted with MIDEAS-AS1 was performed by mass spectrometry. **E** Western blot analysis following RNA pull-down assay indicated that MIDEAS-AS1 interacted with MATR3. **F** RIP assay using Flag antibody showed that MIDEAS-AS1 interacted with MATR3. **G** Colocalization of MIDEAS-AS1 and MATR3 by immunofluorescence following transfected with MIDEAS-AS1-overexpressing vector or control vector. Scale bars, 20 μm. **H** The interaction between the truncated MIDEAS-AS1 and MATR3 was confirmed by RNA pull-down and western blot. Data were shown as mean ± SD. (*p < 0.05, ** *p* < 0.01, *** *p* < 0.001)

It had been suggested that the regulatory mechanisms of lncRNAs were closed associated with their subcellular localization [25]. Therefore, we firstly detected the subcellular localization of MIDEAS-AS1 in TNBC cells. Following isolation of the nuclear and cytoplasmic RNA in MDA-MB-231 and MDA-MB-468 cells, qRT-PCR analysis demonstrated that MIDEAS-AS1 was mainly located in the nucleus (Fig. 3A). Furthermore, FISH assay also obtained the similar results (Fig. 3B), indicating the potential role of MIDEAS-AS1 in transcriptional regulation through acting as a scaffold for TFs [25, 27]. To identify proteins interacted with MIDEAS-AS1, RNA pull-down assay was performed. We incubated MDA-MB-231 cell lysates with biotinylated MIDEAS-AS1 or its antisense RNA transcribed in vitro, and the silver staining and mass spectrometry (MS) identified MATR3 as one of the major proteins in the MIDEAS-AS1 pull-down precipitations (Fig. 3C). Moreover, mass spectrometry analysis found that MIDEAS-AS1 interacted with MATR3 at “DLSAAGIGLLAAATQSLSMPASLGR” and “YQLLQLVEPFGVISNHLILNK” peptide sequences (Fig. 3D). The specific binding between MATR3 protein and MIDEAS-AS1 was further confirmed by RNA pull down following Western blot (Fig. 3E). Then, we performed RNA immunoprotein (RIP) assay using flag antibodies and revealed that MIDEAS-AS1 was significantly enriched in MATR3 immunoprecipitations (Fig. 3F). Meanwhile, RNA FISH technology combined with immunofluorescence analysis demonstrated the co-localization of MIDEAS-AS1 and MATR3 protein in MDA-MB-231 cells (Fig. 3G). To identify the specific binding regions between MIDEAS-AS1 and MATR3, we constructed vectors containing full-length MIDEAS-AS1 or three truncated sequences (Fig. 3H). The RNA pull down assay indicated that deleting the sequence of 217–439 bp (Δ2 vector) could abolish the MIDEAS-AS1-MATR3 interaction, which was consist with the prediction results obtained from catRAPID database (Additional file 1: Fig. S3A). Furthermore, we wonder whether MIDEAS-AS1 could regulate the expression of MATR3. Significantly, overexpression or knockdown of MIDEAS-AS1 shows no effect on the RNA and protein expression levels of MATR3 (Additional file 1: Fig. S3B), indicating that MIDEAS-AS1 was not involved in the post-transcriptional regulation of MATR3. Collectively, these results revealed that MIDEAS-AS1 specifically interacted with MATR3 in TNBC cells.

### MIDEAS-AS1 affects the progression and metastasis of TNBC by promoting NCALD expression

**Fig. 4:**
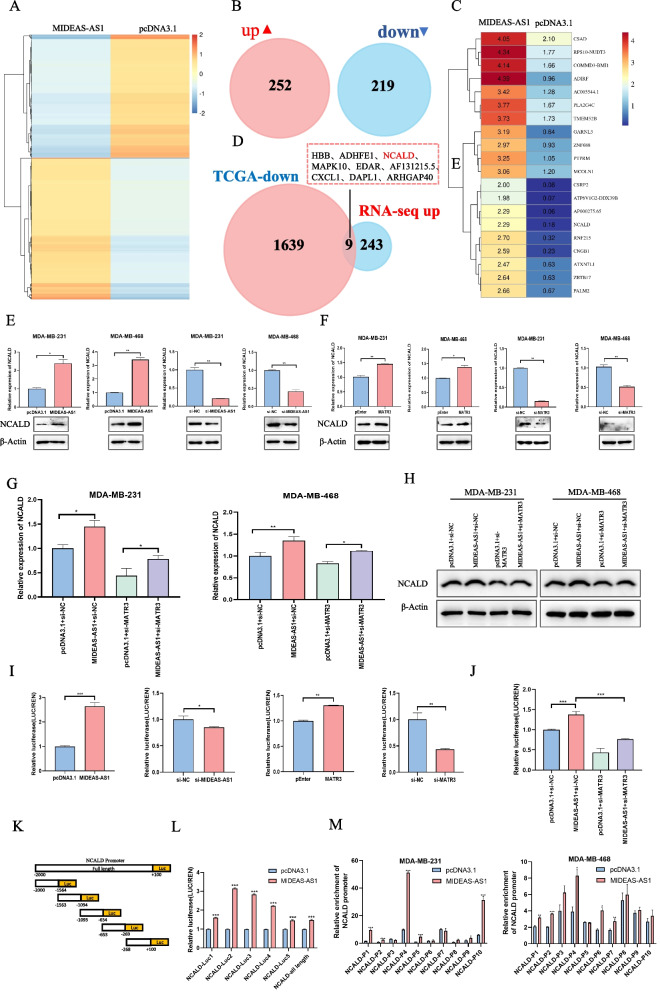
MIDEAS-AS1 activates NCALD expression via recruiting the MATR3 complex to the NCALD promoter. **A** Heat map of differentially expressed gene based on RNA-seq analysis between MIDEAS-AS1-overexpression cells and control cells. **B** There were 471genes differentially expressed between MIDEAS-AS1-overexpression and control (252 up and 219 down) cells (|log2(Fold change) |> 1). **C** Top 20 up-regulated genes were showed. **D** Venn-diagrams showing intersect between down-regulated genes in TCGA data (1648 genes) and up-regulated DEGs after MIDEAS-AS1-overexpressing data (252 genes). **E–F** qRT-PCR and Western blot analysis of NCALD expression in MDA-MB-231cells and MDA-MB-468 cells with knockdown or overexpression MIDEAS-AS1 or MATR3. **G** qRT-PCR analysis of the expression of NCALD in MDA-MB-231 and MDA-MB-468 cells co-transfected with MIDEAS-AS1 overexpression vector or empty vector together with si-MATR3 or si-NC. **H** Western blot analysis of the expression of NCALD in MDA-MB-231 and MDA-MB-468 cells co-transfected with MIDEAS-AS1 overexpression vector or empty vector together with si-MATR3 or si-NC. **I** Luciferase reporter assays validated the binding of MIDEAS-AS1 and MATR3 with NCALD. **J** Luciferase assays in HK293T cells was determined by co-transfected with MIDEAS-AS1-overexpressing vector and si-MATR3. **K-L** Relative luciferase activity of full-length promoter and the other five truncated promoter regions of NCALD in HK293T cells by transfected with MIDEAS-AS1-overexpressing vector. **M** ChIP-qPCR experiments on ten different NCLAD promoter primer using anti-Flag antibody in MDA-MB-231 and MDA-MB-468 cells transfected with MIDEAS-AS1 overexpression plasmid and N-terminal FLAG-tagged MATR3 plasmid. Data were shown as mean ± SD. (*p < 0.05, ** *p* < 0.01, *** *p* < 0.001)

Given the critical role of nuclear matrix-associated protein MATR3 in transcriptional regulation, we wonder whether MIDEAS-AS1 could regulate the expression of downstream genes through interacting with MATR3 to further affect the progression and metastasis of TNBC. We performed an RNA-sequencing analysis on MIDEAS-AS1-overexpressing and control cells, and 471 differentially expressed genes were identified (Fig. 4A-B), including 252 up-regulated and 219 down-regulated genes. Meanwhile, the top 20 up-regulated genes are shown in Fig. 4C. Due to the tumor-suppressive role of MIDEAS-AS1 in TNBC, we integrated the down-regulated genes in breast cancer tissues from TCGA database and the up-regulated genes after MIDEAS-AS1 overexpression in TNBC cells, and then, 9 candidate genes were selected as potential downstream targets of MIDEAS-AS1 (Fig. 4D). It has been reported in the literature that antisense lncRNA may affect the expression of sense gene [28], so we also examined the influence of MIDEAS-AS1 on its sense gene (MIDEAS) by qRT-PCR. However, the results showed that MIDEAS-AS1 did not affect the expression of MIDEAS (Additional file 1: Fig. S4B). Meanwhile, the qRT-PCR results revealed that the expression of AF131215.5, CXCL1, and NCALD was remarkably increased after MIDEAS-AS1 overexpression and reduced after MIDEAS-AS1 knockdown (Additional file 1: Fig. S4A). CXCL1 (C-X-C motif chemokine ligand 1) was the most abundant chemokine secreted by TAMs, and CXCL1 could promote migration, invasion ability, and EMT in breast cancer [29, 30]. AF131215.5 was a lncRNA whose function remained unclear in cancer [31, 32]. Interestingly, NCALD (neurocalcin delta) expression was lower in lung adenocarcinoma tissues [33], and patients with higher NCALD levels exhibited a higher survival rate [34, 35]. Among these genes, we hypothesized that NCALD, the tumor suppressor gene, might be a potential target of MIDEAS-AS1 in breast cancer. Significantly, NCALD expression was markedly downregulated in breast cancer tissues compared with normal tissues (Additional file 1: Fig. S4C-D). Moreover, immunohistochemistry (IHC) showed higher expression of NCALD in normal tissues than breast cancer tissues according to Human Protein Atlas database (Additional file 1: Fig. S4E). These results indicated that NCALD might play a significant tumor suppressor role in breast cancer. Therefore, NCALD was selected as the functional downstream mediator for MIDEAS-AS1-mediated cell migration and metastasis in TNBC. Significantly, the qRT-PCR and Western blot results revealed that the expression of NCALD was remarkably increased after MIDEAS-AS1 or MATR3 overexpression and reduced after MIDEAS-AS1 or MATR3 knockdown in MDA-MB-231 and MDA-MB-468 cells (Fig. 4E-F, Additional file 1: Fig. S4E), indicating the regulatory effect of MIDEAS-AS1 and MATR3 on the expression of NCALD. Furthermore, rescue experiments were performed to demonstrate the causal link between MIDEAS-AS1, MATR3 and NCALD. The qRT-PCR analysis indicated that MIDEAS-AS1 overexpression promoted the expression of NCALD, while MATR3 knockdown could weaken the increased tendency of NCALD expression caused by overexpression of MIDEAS-AS1 in MDA-MB-231 and MDA-MB-468 cells (Fig. 4G). Furthermore, we found that the increasing trend of NCALD protein level caused by MIDEAS-AS1 overexpression could be attenuated by MATR3 knockdown in MDA-MB-231 and MDA-MB-468 cells (Fig. 4H). Then, we further investigated the transcriptional regulation effect of MIDEAS-AS1 and MATR3 on the expression of NCALD. The dual luciferase reporter assay showed that overexpression of MIDEAS-AS1 or MATR3 substantially increased the luciferase activity of NCALD vectors, while knockdown of MIDEAS-AS1 or MATR3 significantly inhibited the luciferase activity of NCALD vectors in HK293T cells (Fig. 4I). Furthermore, rescue experiments revealed that the promoter activity of NCALD was activated by MIDEAS-AS1 overexpression, which was inhibited after co-transfection with si-MATR3 (Fig. 4J). These results indicated that MIDEAS-AS1 could regulate the expression of NCALD through modulating the function of MATR3. To identify the specific binding regions of MIDEAS-AS1 and MATR3 on the NCALD promoter, we ectopically expressed full-length NCALD promoter (−2000 to + 100 bp) as well as five mutants: the truncated NCALD-1 (−2000 to −1564 bp) mutant, the truncated NCALD-2 (−1563 to −1094 bp) mutant, the truncated NCALD-3 (−1093 to −654 bp) mutant, the truncated NCALD-4 (−653 to −269 bp) mutant, and the truncated NCALD-5 (−268 to + 100 bp) mutant (Fig. 4K), and a luciferase assay was performed. We found that the luciferase activity was most significantly increased in −1563 bp to −1094 bp, indicating the presence of positive regulatory elements, which enhanced NCALD transcription in this region (Fig. 4L). In addition, the ChIP followed by qPCR assays was subsequently performed in Flag-MATR3 transfected breast cancer cells with or without MIDEAS-AS1 overexpression to determine the effect of MIDEAS-AS1 on MATR3 recruitment to the NCALD promoter. Consistent with the luciferase assay, MIDEAS-AS1-MATR3 complex was identified to significantly bound to −1563 bp to −1094 bp sites of the NCALD promoter region in MDA-MB-231 and MDA-MB-468 cells (Fig. 4M). These findings suggested that MIDEAS-AS1-MATR3 complex could enhance NCALD transcription by directly binding to its promoter.

### NCALD inhibits TNBC cell proliferation, migration, and invasion in vitro

**Fig. 5:**
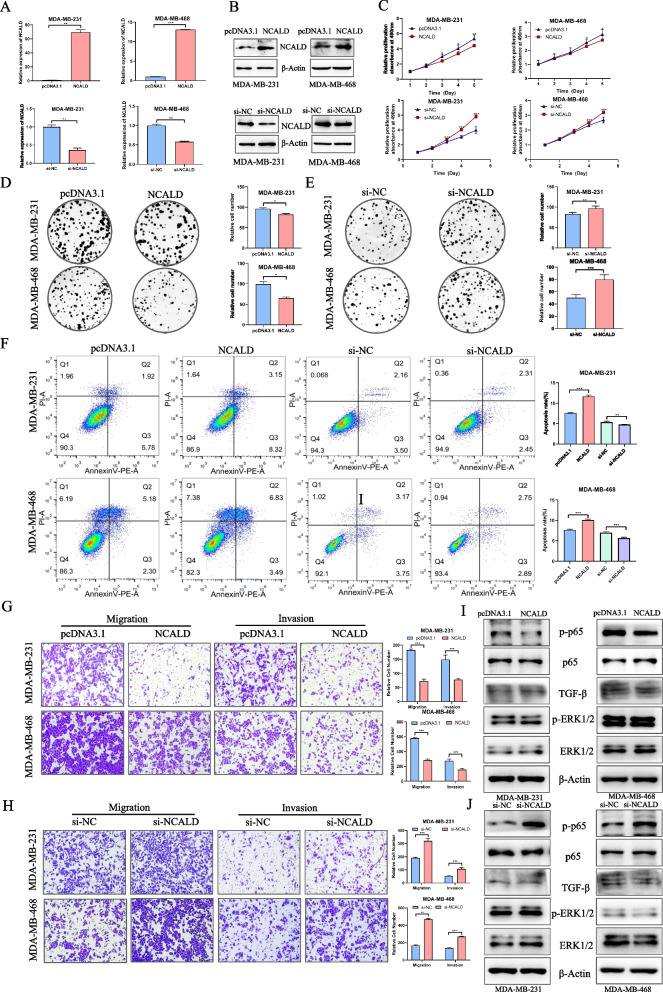
The overexpression of NCALD inhibits TNBC cell proliferation and migration in vitro. **A-B** The efficiency of overexpression and knockdown of NCALD were confirmed by qRT-PCR (**A**) and Western blot (**B**) in MDA-MB-231 and MDA-MB-468 cells. **C–E** The effects of NCALD overexpression and knockdown on the proliferation of MDA-MB-231 and MDA-MB-468 cells were examined by MTT assay (**C**) and colony formation assays (**D-E**). **F** Flow cytometry was performed to measure the effect of NCALD overexpression and knockdown on apoptosis. **G** Transwell and invasion were used to evaluate the motility of MDA-MB-231 and MDA-MB-468 cells transfected with NCALD-overexpressing vector or control vector (**G**) and si-NC or si-MIDEAS-AS1 (**H**). **I** Western blot analysis of p-ERK1/2, p-NF-κB and TNF-β protein levels in MDA-MB-231 and MDA-MB-468 cells transfected with NCALD overexpression plasmid. **J** Western blot analysis of p-ERK1/2, p-NF-κB and TNF-β protein levels in MDA-MB-231 and MDA-MB-468 cells transfected with transfected with si-NC or si-NCALD. Data were shown as mean ± SD. (*p < 0.05, ** *p* < 0.01, *** *p* < 0.001)

Previous studies had found that NCALD was not only involved in cell apoptosis, cell cycle progression and other biological processes in several cancers [36], but also associated with the prognosis of cancers [34, 35]. However, there is no report about the function of NCALD in breast cancer. Our above results revealed downregulated expression of NCALD in breast cancer tissues and breast cancer cells, indicating the tumor-suppressive role of NCALD in breast cancer. To confirm the function of NCALD, we transfected NCALD overexpressing vectors and si-NCALD into MDA-MB-231 and MDA-MB-468 cells, and the overexpression and knockdown efficiency were determined by qRT-PCR and Western blot assays (Fig. 5A-B). The MTT and colony formation assay results indicated that NCALD overexpression reduced TNBC cell proliferation and colony formation abilities (Fig. 5C, D). Additionally, the apoptosis rate was remarkably increased and the number of cells in G1 phase was increased after NCALD overexpression (Fig. 5F, Additional file 1: Fig. S5A). On the other hand, NCALD knockdown led to significantly increased proliferation, colony formation abilities of TNBC cells (Fig. 5C, E). Moreover, the apoptosis rate was decreased and the number of the cells in G1 phase was less in NCALD knockdown cells than that in control cells (Fig. 5F, Additional file 1: Fig. S5A). Additionally, transwell assay and wound healing assay revealed that NCALD overexpression significantly reduced TNBC cell migration and invasion abilities (Fig. 5G, Additional file 1: Fig. S5B), while NCALD knockdown led to increased migration and invasion abilities in TNBC cells (Fig. 5H, Additional file 1: Fig. S5B). These data demonstrated that NCALD served as a suppressive functional factor in TNBC progression.

Previous literature reported that NCALD might be related to ERK1/2 signaling pathway, NF-κB signaling pathway, TGF-β signaling pathway and immune response pathway in ovarian cancer [35]. We further investigated the potential molecular mechanism caused by the change of NCALD in TNBC cells. Western blot analysis indicated that overexpression of NCALD would significantly inhibited phosphorylate p-65, but not TGF-β and, p-ERK1/2 in MDA-MB-231 and MDA-MB-468 cells (Fig. 5I), while NCALD knockdown brought about opposite results (Fig. 5J). Collectively, our results demonstrated that NCALD inhibited TNBC cell proliferation, migration, and invasion by suppressing NF-κB signaling pathways.

### MIDEAS-AS1 regulates TNBC progression through regulating the expression of NCALD

**Fig. 6:**
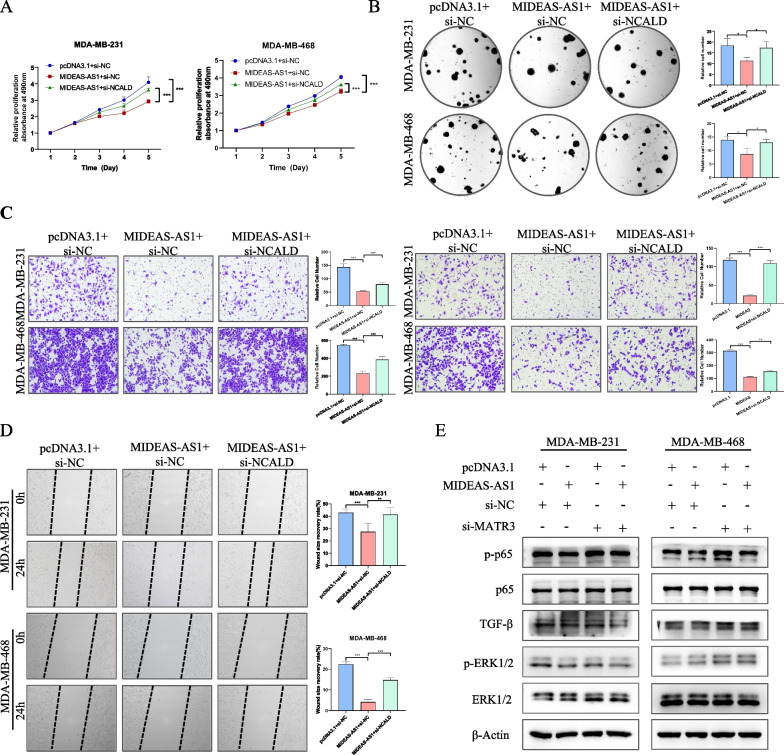
MIDEAS-AS1 exerts its function by regulating the NCALD expression and downstream signaling. **A-B** The effects of co-transfected with MIDEAS-AS1 overexpression vector or empty vector together with si-NCALD or si-NC on the proliferation of MDA-MB-231 and MDA-MB-468 cells by MTT assay (**A**) and colony formation assays (**B**). **C-D** Transwell and wound-healing assays were used to evaluate the motility of MDA-MB-231 and MDA-MB-468 cells of co-transfected with MIDEAS-AS1 overexpression vector or empty vector together with si-NCALD or si-NC. **E** Western blot analysis of the corresponding signaling in MDA-MB-231 and MDA-MB-468 cells co-transfected with MIDEAS-AS1 overexpression vector or empty vector together with si-MATR3 or si-NC. Data were shown as mean ± SD. (*p < 0.05, ** *p* < 0.01, *** *p* < 0.001)

Our previous results have showed that the association between MIDEAS-AS1 and MATR3 played significant role in initiating NCALD transcription. To further confirm whether MIDEAS-AS1 exerts tumor-suppressive functions via modulating NCALD, we co-transfected si-NCALD and MIDEAS-AS1-overexpressed plasmid in TNBC cell lines. The MTT and colony formation experiments indicated that NCALD knockdown remarkably rescued the proliferation ability of MDA-MB-231 and MDA-MB-468 cells inhibited by the overexpression of MIDEAS-AS1 (Fig. 6A-B). Moreover, the transwell and wound healing assays showed that NCALD knockdown could partially recover the cell migration and invasion abilities reduced by MIDEAS-AS1-overexpression (Fig. 6C-D). Moreover, Western blot analysis revealed that NF-κB pathway-related proteins were decreased after overexpression of MIDEAS-AS1, and that was increased after co-transfection of MIDEAS-AS1 overexpression plasmid and si-MATR3 (Fig. 6E). Taken together, MIDEAS-AS1 associates with MATR3 to initiate NCALD transcription and inhibits NF-κB signaling pathway, which further affects the TNBC progression.

### MIDEAS-AS1 overexpression inhibits TNBC progression and metastasis in vivo

**Fig. 7:**
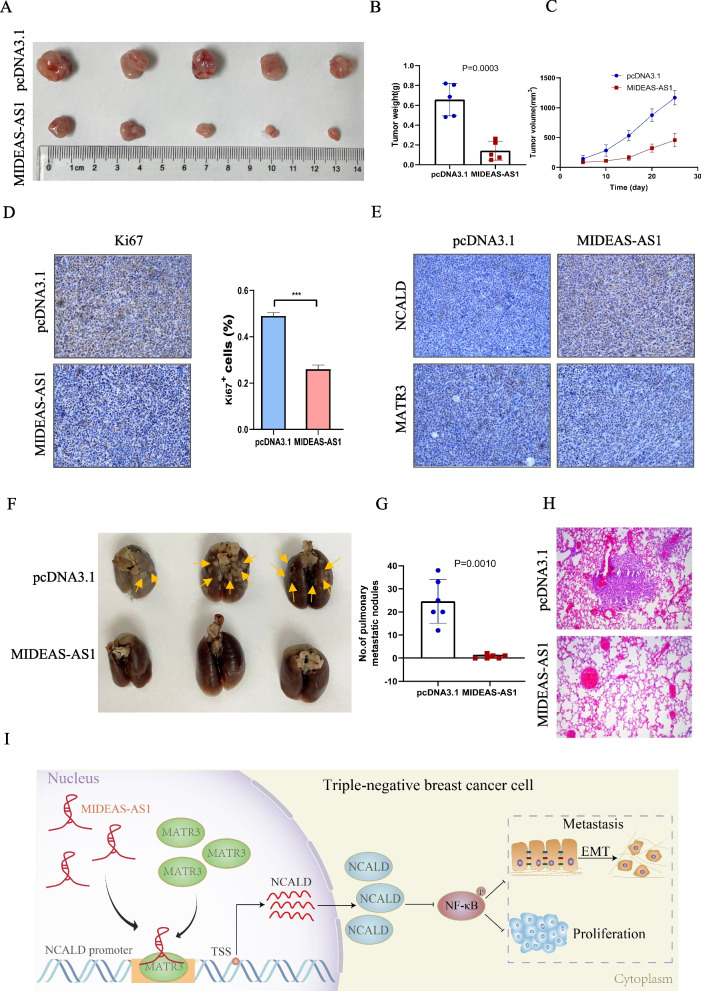
MIDEAS-AS1 overexpression inhibits tumor formation in nude mice xenograft models. **A** MDA-MB-231 cells were stably transfected with the MIDEAS-AS1 overexpression plasmid or control plasmid and inoculated subcutaneously into nude mice. Compared with the control plasmid group, MIDEAS-AS1 overexpression inhibited tumor growth. **B-C** Tumor weight (**B**) Tumor volume (mm^3^) (**C**) were significantly decreased in the MIDEAS-AS1 overexpression group. **D** Immunohistochemistry with a Ki67-specific antibody was performed in the tumor. The results showed that MIDEAS-AS1 overexpression led to reduced expression of Ki67. Scale bars, 50 μm. **E** Representative images MATR3, NCALD staining in the tumor. Immunohistochemical staining showed that MIDEAS-AS1 overexpression led to increased expression of NCALD, while MATR3 was not affected. Scale bars, 50 μm. **F-G** Stably transfected MDA-MB-231 cells were injected into the tail veins of nude mice (*n* = 5). Representative images of lungs (**F**) and HE staining of lungs (**H**) isolated from mice. MIDEAS-AS1 overexpression resulted in a decreased number of lung metastatic colonies (**G**). **I** Schematic diagram depicting mechanisms involved in the effect of MIDEAS-AS1. Data were shown as mean ± SD. (*p < 0.05, ** *p* < 0.01, *** *p* < 0.001)

In order to further evaluate the biological function of MIDEAS-AS1 in vivo, a subcutaneous xenograft model was first constructed. The MDA-MB-231 cells stably transfected with MIDEAS-AS1 overexpressing vectors or control vectors were subcutaneously injected to the flanks of nude mice. The result showed that the tumor weight and tumor volume were significantly inhibited in xenografts of MIDEAS-AS1 overexpressing group compared with those in the control group (Fig. 7A–C). Furthermore, immunohistochemistry (IHC) assays confirmed that MIDEAS-AS1 overexpression caused decreased Ki67 expression and increased NCALD expression, but did not affect MATR3 expression (Fig. 7D-E). In addition, we further constructed pulmonary metastasis model through intravenously injecting MDA-MB-231 cells stably expressing MIDEAS-AS1 or control vectors to compare the metastatic abilities in vivo. The results showed that mice injected with MIDEAS-AS1-overexpressing TNBC cells had no or fewer metastatic foci compared with the control group (Fig. 7F–H). Together, these results indicated that MIDEAS-AS1 played a critical role in inhibiting TNBC progression and metastasis.

## Discussion

Breast cancer is one of the most common malignancies among women worldwide and is the major cause of most cancer-related deaths. There are several explanations for the high mortality rate of breast cancer, with metastasis of vital organs thought to be the main cause [37]. As reported in the literature, TNBC is the most challenging subtype of breast cancer with higher rates of relapse and greater metastatic potential compared to other breast cancer subtypes [4, 38, 39]. Despite the treatment methods for patients with metastatic breast cancer are complicated, the curative effects are unsatisfactory in the clinical practice. Therefore, it is of great significance to investigate the molecular mechanism of TNBC metastasis and identify novel prognostic predictors for accurate diagnosis and prediction of prognosis.

It is well known that lncRNA is abnormally expressed in many different types of cancer and is involved in the regulation of tumor development and progression [40–42]. Recently, various studies have focused on the functions and regulations of lncRNAs to search novel diagnostic and therapeutic targets for cancer treatment. In this study, we explored the potential role and molecular mechanism of MIDEAS-AS1 in TNBC progression. We determined that MIDEAS-AS1 was significantly downregulated in TNBC tissues compared to the non-TNBC tissues, and low expression of MIDEAS-AS1 was associated with poor prognosis in TNBC. Functional studies revealed that MIDEAS-AS1 suppressed proliferation, migration and invasion, metastasis and promoted apoptosis of TNBC cells in vitro, indicating a tumor suppressor role in TNBC. Moreover, MIDEAS-AS1 inhibited TNBC tumor progression and lung metastasis in vivo by xenograft model. However, the regulatory mechanism of MIDEAS-AS1 involved in TNBC progression was still unclear and worthy of further exploration.

It is reported that the localization of lncRNAs within the cell is the primary determinant of their molecular functions [43]. Increasing evidence suggests that cytoplasmic lncRNAs can regulate gene expression by modulating mRNA stability, translation process or participating in mRNA post-transcriptional regulation as ceRNAs [19, 44]. Meanwhile, the nuclear lncRNAs also participate in several biological processes, including chromatin organization, and transcriptional and post-transcriptional gene expression, and acting as structural scaffolds of nuclear domains [24]. Here, we found that MIDEAS-AS1 was mainly located in the nucleus based on cell cytoplasmic/nuclear fractionation and RNA FISH assays. Previous studies have reported that lncRNAs could play important role in transcription by recruiting corresponding proteins [45, 46]. Therefore, MIDEAS-AS1 might exert its function by recruiting transcription complexes and further enhance or inhibit gene transcription. Then, RNA pull-down followed by mass spectrometry showed the binding potential between MATR3 and MIDEAS-AS1, which was further confirmed by RIP assay. Moreover, we also found that MIDEAS-AS1 and MATR3 were co-localization in TNBC cells through RNA FISH technology combined with immunofluorescence. Subsequently, we identified the specific binding region between MIDEAS-AS1 and MATR3. However, we found that the expression level of MIDEAS-AS1 did not affect the RNA and protein levels of MATR3, making us speculating that MIDEAS-AS1 might regulated the function of MATR3 in TNBC cells.

It is reported that MATR3 is an abundant nuclear protein that binds with DNA and RNA [47, 48], allowing MATR3 to play crucial roles in RNA splicing and gene transcription [49–51]. To further explore the downstream genes regulated by the MIDEAS-AS1-MATR3 complex, we analyzed the RNA-seq results and 471 DEGs were revealed. Following integration with TCGA database, we finally identified 9 candidate genes as potential direct downstream targets of MIDEAS-AS1 and MATR3. NCALD, a member of the neuronal calcium sensors protein family [52], caught our attention due to the remarkable changes after MIDEAS-AS1 overexpression or knockdown. Studies have found that NCALD was associated with the prognosis of several cancers, including non-small cell lung cancer, ovarian cancer, colorectal cancer, indicating its clinical potential as a prognostic biomarker [35, 36]. However, the function role and molecular mechanism of NCALD in breast cancer has not been reported. In this study, we found that NCALD was downregulated in breast cancer tissues. Moreover, the expression of NCALD could be regulated by MATR3 in TNBC cells. Significantly, rescue experiment revealed that MATR3 knockdown attenuated the increasing trend of NCALD expression caused by MIDEAS-AS1 overexpression in TNBC cells. Subsequently, dual luciferase reporter assays and ChIP-qPCR indicated that MIDEAS-AS1 and MATR3 complex could bind to NCALD promoter to regulate NCALD transcription.

We further investigated whether NCALD modulate the functional effect of MIDEAS-AS1 in TNBC. Functional experiments revealed that NCALD overexpression inhibited cell proliferation, migration, and invasion in TNBC cells, suggesting the suppressive functional role of NCALD in TNBC progression. Previous study has reported that the TGF-β, NF-κB and ERK signaling pathways were associated with NCALD expression in epithelial ovarian cancer [35]. Interestingly, Western blot analysis showed that overexpression of NCALD inhibited the expression of the NF-κB pathway-associated proteins, indicating that NCALD inhibited progression of TNBC possibly by suppressed NF-κB signaling pathway. Furthermore, the rescue experiments showed that NCALD knockdown could partially rescue the proliferation and migration ability of TNBC cells inhibited by overexpression of MIDEAS-AS1. Western blot analysis also revealed that MIDEAS-AS1 and MATR3 could not only affect the NCALD expression, but also significantly modulate the NF-κB signaling pathway. Therefore, our study revealed that MIDEAS-AS1 regulated TNBC progression by recruiting the MATR3 to initiate NCALD transcription and suppressing NF-κB signaling pathways (Fig. 7I).

## Conclusions

In this study, we identified MIDEAS-AS1 as a novel tumor suppressor in TNBC, and higher expression of MIDEAS-AS1 was associated with better prognosis of TNBC patients. Mechanistically, we revealed that MIDEAS-AS1 inhibited TNBC progression through activating NCALD transcription via recruiting MATR3. Our study illustrates a novel mechanism involved in TNBC progression and suggests that MIDEAS-AS1 might serve as a prognostic biomarker for TNBC patients.

### Supplementary Information

**Additional file 1.** Supplementary figures and tables.

## Data Availability

The data that support the findings of this study are available on request from the corresponding author upon reasonable request.

## Abbreviations

TNBC: Triple-negative breast cancer
lncRNAs: Long non-coding RNAs
RIP: RNA immunoprecipitation
ChIP: Chromatin immunoprecipitation
OS: Overall survival
PFS: Progression-free survival
ceRNA: Competing endogenous RNAs
GEO: Gene Expression Omnibus
TCGA: The Cancer Genome Atlas
