# Supplementary material for: Long non-coding RNA MIDEAS-AS1 inhibits growth and metastasis of triple-negative breast cancer via transcriptionally activating NCALD
Source: Breast Cancer Res. 2023 Sep 28;25:109. doi: 10.1186/s13058-023-01709-1 (PMC10540452; doi:10.1186/s13058-023-01709-1)
Supplement: Supplementary file 1 — Additional file 1. Supplementary figures and tables. [file 13058_2023_1709_MOESM1_ESM.pdf]

Supplemental Information

Supplementary Figures

Fig.S1 MIDEAS-AS1 is lowly expressed in TNBC. (A) Heat map shows the significantly expressed lncRNAs in breast cancer samples compared to normal tissues according to The Cancer Genome Atlas (TCGA) database. The red shades represent high expression, and blue shades represent low expression. (B) Schematic view of the chromosomal location of MIDEAS-AS1. (C) The protein-coding ability of MIDEAS-AS1 is predicted by CPC2 (http://cpc2.gao-lab.org). (D)The expression level of MIDEAS-AS1 in different subtypes of breast cancer tissues by using GSE21653 database. Statistical analysis was performed with the R language. Data were shown as mean ± SD. (*p<0.05, **p<0.01, ***p<0.001).

Fig.S2 Knock down of MIDEAS-AS1 contributes to TNBC cell proliferation and migration in vitro. (A-B) The efficiency of MIDEAS-AS1 overexpression (A) and knockdown (B) were examined by qRT-PCR. (C) Flow cytometry was performed to measure the effect of MIDEAS-AS1 overexpression and knockdown on cell cycle in vitro. (D) Wound-healing assays were used to evaluate the motility of MDA-MB-231 and MDA-MB-468 cells transfected with MIDEAS-AS1-overexpressing vector or control vector and si-NC or si-MIDEAS-AS1. Data were shown as mean ± SD. (*p<0.05, **p<0.01, ***p<0.001).

Fig.S3 Effect of overexpression or knockdown of MIDEAS-AS1 on MATR3. (A)

Interaction sites of MIDEAS-AS1 and MATR3 genes were analyzed from the catRAPID database. (B) The influence of knockdown or overexpression MIDEAS-AS1 on the expression level of MATR3 in MDA-MB-231 and MDA-MB-468 cells was determined by qRT-PCR and western blot analysis.

Fig.S4 NCALD is downregulated in breast cancer tissues. (A) qRT-PCR analysis was used to verify the relative expression of 9 candidate genes in MDA-MB-231 and MDA-MB-468 cells with knockdown or overexpression MIDEAS-AS1. (B) Effect of overexpression and knockdown of MIDEAS-AS1 on expression of its sense gene (MIDEAS) by qRT-PCR assay in MDA-MB-231 and MDA-MB-468 cells. (C) Relative expression of NCALD in tumor tissue and adjacent normal breast tissues from TCGA data. (D) Relative expression of NCALD in tumor tissue and adjacent normal breast tissues from a BCIP cohort. (E) Expression of NCALD in tumor and normal breast tissues detected by IHC from Human Protein Atlas (https://www.proteinatlas.org/). (F) The efficiency of knockdown and overexpression of MATR3 was confirmed by qRT-PCR and Western blot in MDA-MB-231 and MDA-MB-468 cells. Data were shown as mean ± SD. (*$p<0.05$, **$p<0.01$, ***$p<0.001$).

Fig.S5 Knock down of NCALD promotes TNBC cell proliferation and migration in vitro. (A) Flow cytometry was performed to measure the effect of NCALD overexpression and knockdown on cell cycle in vitro. (B) Wound-healing assays were

used to evaluate the motility of MDA-MB-231 and MDA-MB-468 cells transfected with NCALD-overexpressing vector or control vector and si-NC or si-NCALD. Data were shown as mean ± SD. (*$p<0.05$, **$p<0.01$, ***$p<0.001$).

Supplementary Tables

Table S1. si-RNA and plasmids used for transfection.

Name	Sequence (5'-3')
MIDEAS-AS1	F CGGAATTCCCCCTCGGCTGCGGGGTAG
	R CGGGATCCTGCCAAAATGAACACTACTGTCATG
MIDEAS-AS1(for organoid)	F GCTCTAGACCCTCGGCTGCGGGGTAG
	R CGGGATCCTGCCAAAATGAACACTACTGTCATG
NCLAD	F CCCTCGAGATGGGGAAACAGAACAGCAAGCT
	R GCTCTAGATCAGAACTGGCCGGCACTGCTC
MATR3	F CGCAAATGGGCGGTAGGCGTG
	R CCTCTACAAATGTGGTATGGC
NCALD-Luc1	F GGGGTACCCTGGTTTGGGAAAAGGTATGGC
	R CCCTCGAGTTCCCTTCTTTCCCCAACCCC
NCALD-Luc2	F GGGGTACCTGAGTGCTAATAGGTGTGGGAT
	R CCCTCGAGTTCACGTTTAATGTGAACTCTTC
NCALD-Luc3	F GGGGTACCCTTCTGGCTTCTCTTGGGGGCA
	R CCCTCGAGAGAGAATCTAATCTAATTGTTA
NCALD-Luc4	F GGGGTACCCGGGGTCGGGCCTAGTATTAAG
	R CCCTCGAGGCCTTCCTCGTTGTAATGTTTGC
NCALD-Luc5	F GGGGTACCCAGGTCACGGTGCCTTTGCAGCCT
	R CCCTCGAGTGCTTTTGTCTAACTAGTGCTTTC
NCALD-all length (Luc)	F GGGGTACCCTGGTTTGGGAAAAGGTATGGCA
	R CCCTCGAGTGCTTTTGTCTAACTAGTGCTTTC
MIDEAS-AS1-Sense	F TAATACGACTCACTTACATAGGGCCCTCGGCTGCGGGGTAG
	R TGCCAAAATGAACACTACTGTCATG
MIDEAS-AS1-Antisense	F TAATACGACTCACTTACATAGGGTGCCAAAATGAACACTACTGTCATG
	R CCCTCGGCTGCGGGGTAG
MIDEAS-AS1-Δ1-sense	F1 TAATACGACTCACTATAGGGCCTGCCAATGATCTCTGCCTCCA
MIDEAS-AS1-Δ2	R2 AGTTCTCAATGTTGCCTCATGGGCGCATCTTAGAGGCTGT
MIDEAS-AS1-Δ2-sense	F2 ACAGCCTCTAAGATGCGCCCATGAGGCAACATTGAGAA
MIDEAS-AS1-Δ3	R3 TGCTGTGCTGCCAGCAGCCAGCTCA
si-MIDEAS-AS1	Sense CCAUUUCUCCACUUUGGAATT
	Antisense UUCCAAAGUGGAGAAAUGGTT
si-MATR3	Sense GCCAGUGAGAGUUCAUUUATT
	Antisense UAAAUGAACUCUCACUGGCTT
si-NCALD	Sense UUCUCCGAACGUGUCACGUTT
	Antisense ACGUGACACGUUCGGAGAA
si-NC	Sense UUCUCCGAACGUGUCACGUTT
	Antisense ACGUGACACGUUCGGAGAATT

Table S2. Primers used for qRT-PCR.

Name	Sequence (5'-3')
MIDEAS-AS1	F TTCCCATCCCAGAAGATAAAG
	R TTGCCTCATTGCTGTGCTG
U6	F CTCGCTTCGGCAGCACA
	R AACGCTTCACGAATTTGCGT
GAPDH	F GTCTCCTCTGACTTCAACAGCG
	R ACCACCCTGTTGCTGTAGCCAA
MATR3	F CAGCAGTCTACAAATCCAGCACC
	R CTGCATGTGTCTAGGTCCTTGC
NCALD	F TCATCATCGCCTTGAGTGT
	R TTGACTCATCTTCAGGCATT
HBB	F AGGAGAAGTCTGCCGTTACTG
	R CCGAGCACTTTCTTGCCATGA
ADHFE1	F TGGACTTTCACCTTCTGGGAA
	R GGAGAGGTTCTTGTCTGTCATCA
MAPK10	F CAGATGGAATTAGACCATGAGCG
	R TCAATGTGCAATCAGACTTGACT
EDAR	F CGAGCGGAATACTCAAACTGCG
	R TAGTCCTCGTCTTTGGTGCCGT
AF131215.5	F AGCCAGGGGAACAAGGTTAAAAG
	R ATCTGATCACCTTACCACCTCCA
CXCL1	F AGCTTGCCTCAATCCTGCATCC
	R TCCTTCAGGAACAGCCACCAGT
DAPL1	F AGAAATTGGCACCTTGGAAAGACA
	R GCTTCTCCAGTGCGTCATTCAG
ARHGAP40	F AGCCTTCAACATGGACTCTGC
	R TTTGGGGACGGTAAACTTCGG
NCALD-P1	F CTGGTTTGGGAAAAGGTATGG
	R TATCCATTTATCAGTTGACAGACA
NCALD-P2	F TGACATCGTTTGTTCATAAAGAG
	R CCAACTTCCAGTGACCACTTATC
NCALD-P3	F TGGGGAAAGAAGGGAATGA
	R GTGATTTTTCTTTAATGATCTCTAC
NCALD-P4	F CAATAAAGAAAAGTAGATAAAGTGG
	R TTCACGTTTAATGTGAACTCTTCTA
NCALD-P5	F TCTGGCTTCTCTTGGGGGCAG
	R GACAAACTGAGAAGGGCACAA
NCALD-P6	F CTTTTATCCTTCAGGGACATTTG
	R CAGGGGTGTCCAGCTTTGTA
NCALD-P7	F TTAGATTAGATTCTCTCGGGGTCGG
	R TTAGCAGGTTAGCCCAGCAT
NCALD-P8	F TGATGGTTTAACAGCCTCCTAGA

	R GCCTTCCTCGTTGTAATGTTTG
NCALD-P9	F TGCCTTTGCAGCCTTGTTA
	R CCAGCACCTGTTTCCTGTCTT
NCALD-P10	F ATTGGGTGAATGAATTGACAAGA
	R GGCTGCTTTTGTCTAACTAGTGCTT
MIDEAS	F AGCGGAAAAGTGTATTGGCCT
	R GCTCAGCAGGGTTCTGTTCA
Actin	F CATGTACGTTGCTATCCAGGC
	R CTCCTTAATGTCACGCACGAT

Figure S1

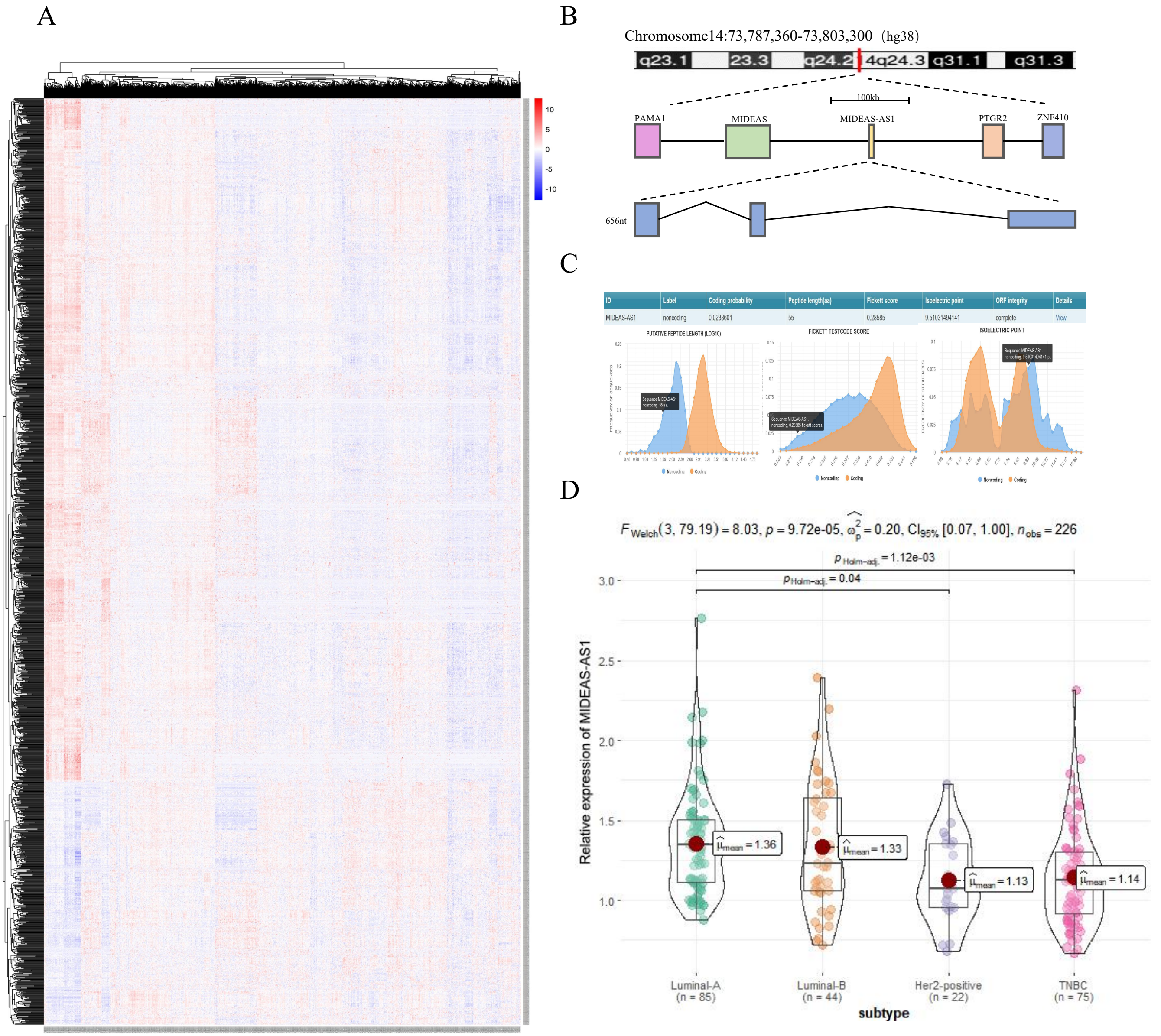

A
B
Chromosome14:73,787,360-73,803,300 (hg38)
q23.1
23.3
q24.2
4q24.3
q31.1
q31.3
100kb
PAMA1
MIDEAS
MIDEAS-AS1
PTGR2
ZNF410
656nt
C
ID
Label
Coding probability
Peptide length(aa)
Fickett score
Isoelectric point
ORF integrity
Details
MIDEAS-AS1
noncoding
0.0238601
55
0.28585
9.51031494141
complete
View
PUTATIVE PEPTIDE LENGTH (LOG10)
FICKETT TESTCODE SCORE
ISOELECTRIC POINT
Noncoding
Coding
D
$F_{Welch}(3, 79.19) = 8.03$, $p = 9.72e-05$, $\widehat{\omega_p^2} = 0.20$, $CI_{95\%}$ [0.07, 1.00], $n_{obs} = 226$
$p_{Holm-adj.} = 1.12e-03$
$p_{Holm-adj.} = 0.04$
Relative expression of MIDEAS-AS1
$\hat{\mu}_{mean} = 1.36$
$\hat{\mu}_{mean} = 1.33$
$\hat{\mu}_{mean} = 1.13$
$\hat{\mu}_{mean} = 1.14$
Luminal-A (n = 85)
Luminal-B (n = 44)
Her2-positive (n = 22)
TNBC (n = 75)
subtype

Figure S2

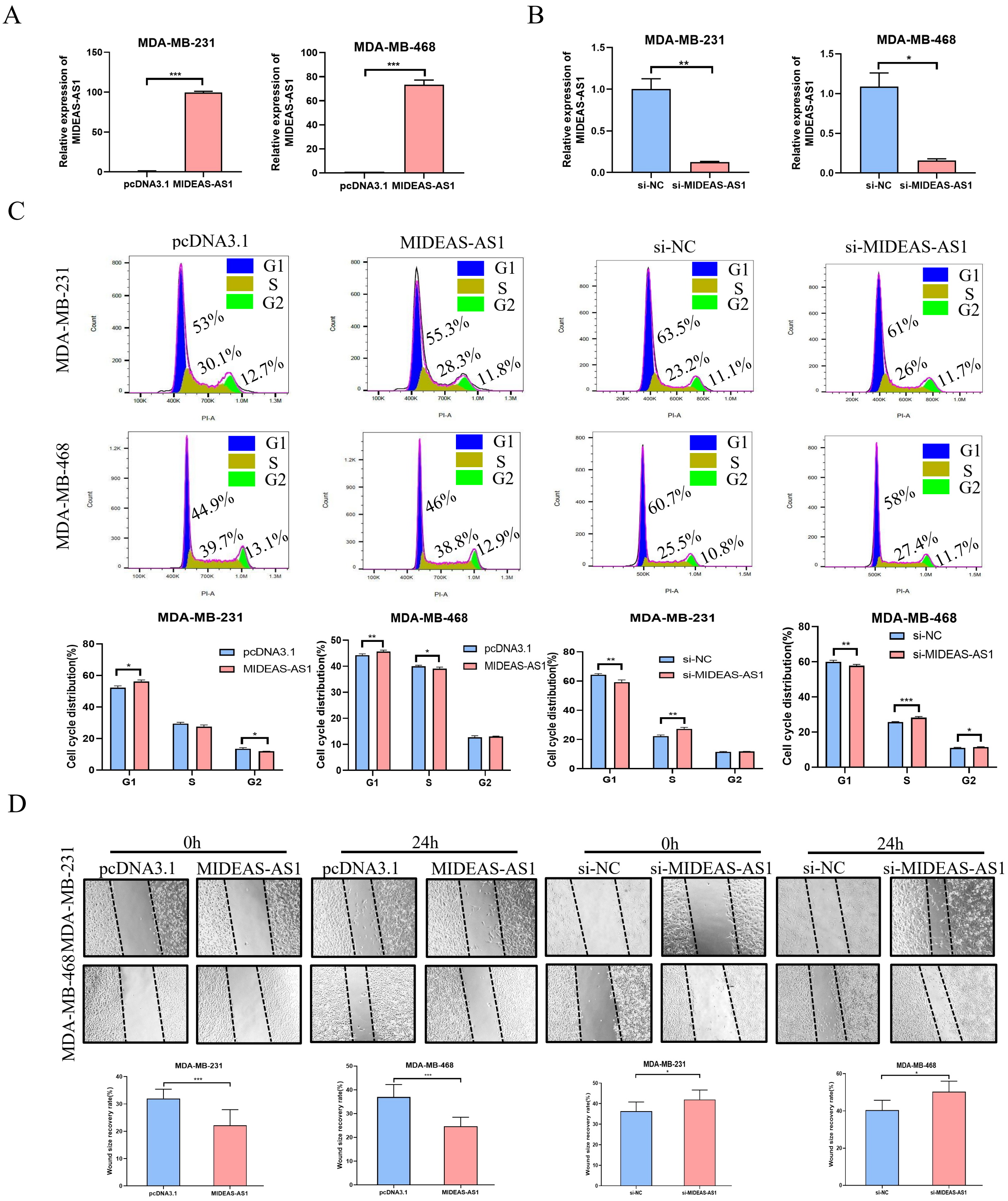
A
MDA-MB-231
Relative expression of MIDEAS-AS1
pcDNA3.1 MIDEAS-AS1
MDA-MB-468
B
MDA-MB-231
si-NC si-MIDEAS-AS1
MDA-MB-468
C
pcDNA3.1
MIDEAS-AS1
si-NC
si-MIDEAS-AS1
MDA-MB-231
MDA-MB-468
G1
S
G2
Count
PI-A
53%
30.1%
12.7%
55.3%
28.3%
11.8%
63.5%
23.2%
11.1%
61%
26%
11.7%
44.9%
39.7%
13.1%
46%
38.8%
12.9%
60.7%
25.5%
10.8%
58%
27.4%
11.7%
Cell cycle distribution(%)
D
0h
24h
Wound size recovery rate(%)

Figure S3

A

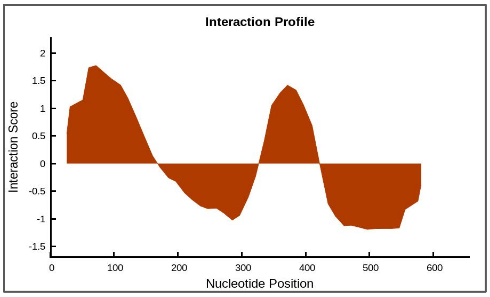
Interaction Profile
Interaction Score
Nucleotide Position
2
1.5
1
0.5
0
-0.5
-1
-1.5
0
100
200
300
400
500
600

B

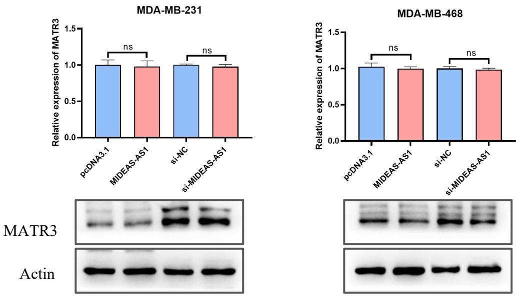
MDA-MB-231
MDA-MB-468
Relative expression of MATR3
ns
pcDNA3.1
MIDEAS-AS1
si-NC
si-MIDEAS-AS1
MATR3
Actin

Figure S4

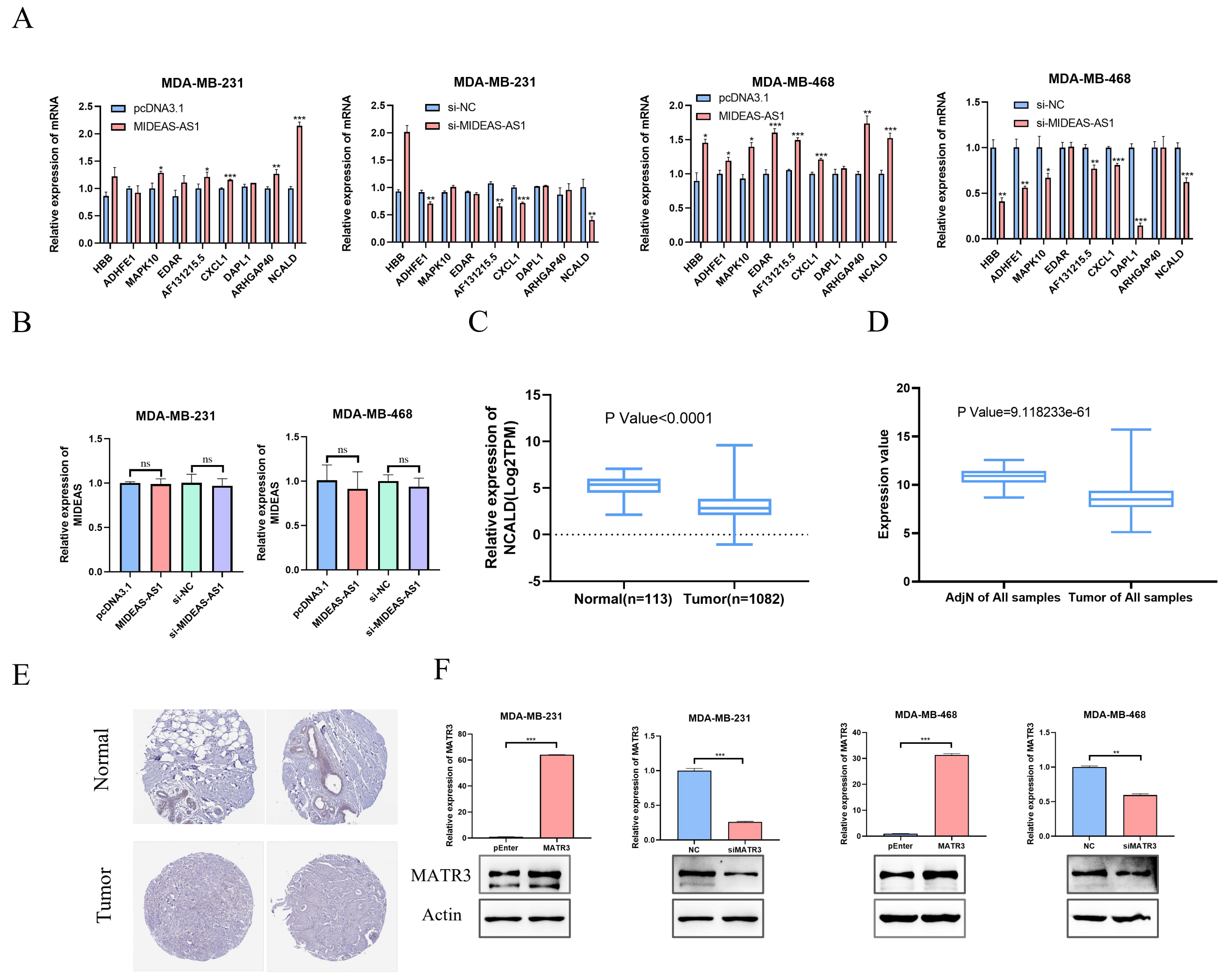
A
MDA-MB-231
pcDNA3.1
MIDEAS-AS1
si-NC
si-MIDEAS-AS1
MDA-MB-468
Relative expression of mRNA
HBB
ADHFE1
MAPK10
EDAR
AF131215.5
CXCL1
DAPL1
ARHGAP40
NCALD
B
Relative expression of MIDEAS
pcDNA3.1
MIDEAS-AS1
si-NC
si-MIDEAS-AS1
ns
C
P Value<0.0001
Relative expression of NCALD(Log2TPM)
Normal(n=113)
Tumor(n=1082)
D
P Value=9.118233e-61
Expression value
AdjN of All samples
Tumor of All samples
E
Normal
Tumor
F
Relative expression of MATR3
pEnter
MATR3
NC
siMATR3
MATR3
Actin

Figure S5

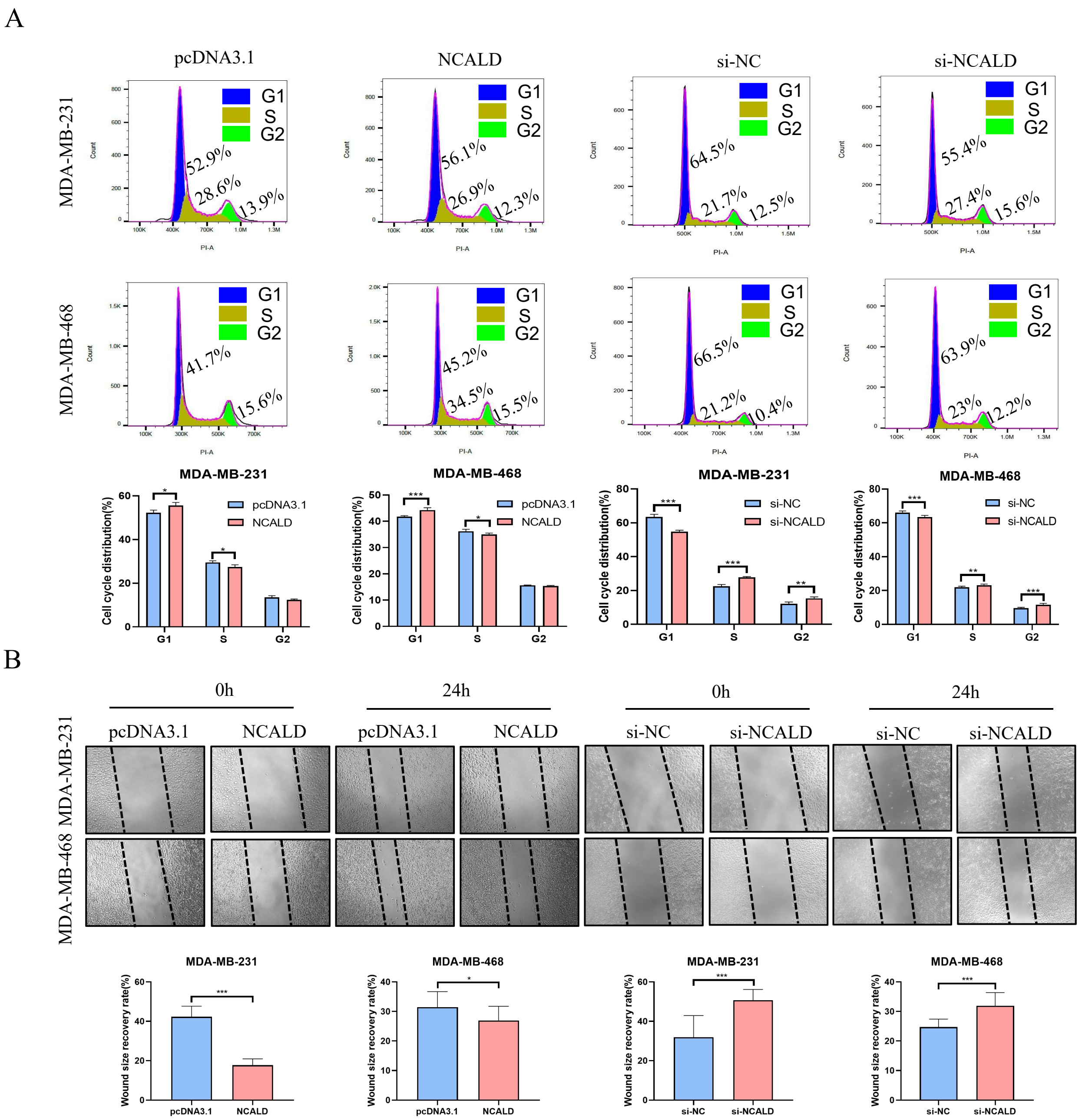
A
pcDNA3.1
NCALD
si-NC
si-NCALD
MDA-MB-231
MDA-MB-468
G1
S
G2
52.9%
28.6%
13.9%
56.1%
26.9%
12.3%
64.5%
21.7%
12.5%
55.4%
27.4%
15.6%
41.7%
15.6%
45.2%
34.5%
15.5%
66.5%
21.2%
10.4%
63.9%
23%
12.2%
Count
PI-A
Cell cycle distribution(%)
B
0h
24h
Wound size recovery rate(%)
